# The compensatory role of T cells from lymph nodes in mice with splenectomy

**DOI:** 10.1111/jcmm.18363

**Published:** 2024-05-21

**Authors:** Xiaobin Liu, Yae Sun, Yanhong Su, Yang Gao, Tianzhe Zhang, Qianhao Wang, Xiaoran Zhang, Dan Zhang, Chenming Sun, Jun Li, Zongfang Li, Baojun Zhang

**Affiliations:** ^1^ Department of Pathogenic Microbiology and Immunology, School of Basic Medical Sciences Xi'an Jiaotong University Xi'an Shaanxi China; ^2^ Department of Medical Immunology, College of Basic Medical Sciences Yan'an University Yan'an Shaanxi China; ^3^ Department of Otolaryngology Head and Neck Surgery The First Affiliated Hospital of Xi'an Jiaotong University Xi'an Shaanxi China; ^4^ Institute of Infection and Immunity, Translational Medicine Institute Xi'an Jiaotong University Health Science Center Xi'an Shaanxi China; ^5^ Key Laboratory of Environment and Genes Related to Diseases Xi'an Jiaotong University Xi'an Shaanxi China; ^6^ Department of Emergency Shaanxi Provincial People's Hospital Xi'an Shaanxi China; ^7^ National‐Local Joint Engineering Research Center of Biodiagnosis & Biotherapy, The Second Affiliated Hospital Xi'an Jiaotong University Xi'an Shaanxi China; ^8^ Shaanxi Provincial Clinical Medical Research Center for Liver and Spleen Diseases, CHESS‐Shaanxi consortium, The Second Affiliated Hospital Xi'an Jiaotong University Xi'an Shaanxi China

**Keywords:** homeostasis, immune response, lymph nodes, splenectomy, T cell

## Abstract

The spleen is a vital organ for the immune system, while splenectomy may be necessary for various reasons. However, there is limited research on the impact of splenectomy on T cell function in peripheral lymph nodes as a compensatory mechanism in preventing infections. This study aimed to investigate the characteristics and function of CD8^+^ and CD4^+^ T cells in different peripheral lymph nodes during viral infection using a well‐established splenectomy model. The results revealed that splenectomy caused an increase in CD8^+^GP33^+^ T cells in the mesenteric lymph nodes (MLN). Moreover, we demonstrated that splenectomy resulted in an increase of effector KLRG1^+^ T cells in the MLN. Additionally, the number of CD4^+^ cytotoxic T cells (CD4 CTLs) was also elevated in the peripheral lymph nodes of mice with splenectomy. Surprisingly, aged mice exhibited a stronger compensatory ability than adult mice, as evidenced by an increase in effector CD8^+^ T cells in all peripheral lymph nodes. These findings provide compelling evidence that T cells in MLN play a crucial role in protecting individuals with splenectomy against viral infections. The study offers new insights into understanding the changes in the immune system of individuals with splenectomy and highlights the potential compensatory mechanisms involved by T cells in peripheral lymph nodes.

## 1 INTRODUCTION

The spleen, a crucial immune organ located in the abdomen, may require removal through splenectomy due to various reasons such as spleen rupture, ectopic spleen, local infection and tumour. Patients who have undergone splenectomy are more vulnerable to infectious diseases due to their weakened immune function, including susceptibility to Streptococcus, Haemophilus influenza, and other pathogens.^1^, ^2^, ^3^ Recent studies have indicated that COVID‐19 patients with splenectomy are at a higher risk of hospitalization and death. However, the risk of infection in patients who have undergone splenectomy is not increased.^4^ Additionally, splenectomy has minimal effects on antiviral immune responses in a mouse model and the effector T cells responded normally to systemic infection in lymph nodes.^5^, ^6^ Lymph nodes, along with the spleen, act as secondary lymphoid tissue and serve as sites for naïve T cells, which can be activated upon encountering antigen presentation from antigen‐presenting cells (APCs).^7^ Therefore, lymph nodes may partially compensate for the immune function of the spleen to protect against infection.

The spleen contains various immune cell populations, including CD4^+^ and CD8^+^ T cells, which are crucial for immune response of anti‐infection.^1^ CD8^+^ T cells are essential for the efficient control of several viral infections, such as coronavirus and herpes viruses.^8^ When bacteria or viruses enter the spleen, primed CD8^+^ cytotoxic T cells, activated by APCs, directly lyse infected cells and produce antiviral cytokines to clear of pathogens.^9^ In contrast to CD8^+^ T cells, the role of CD4^+^ T cells is more complex. CD4^+^ T cells are best known for their ability to assist in B cell and CD8^+^ T cell responses. Naïve CD4^+^ T cells can differentiate into effector populations upon recognizing the antigens presented by MHC class II molecules on activated APCs. Primarily, the CD4^+^ T cells differentiate into the Th1‐type phenotype and produce large amounts of IFNγ in response to infection.^10^ Besides, CD4^+^ T cells can differentiate into T follicular helper (Tfh) cells to assist B cells in generating antibody responses once they enter B cell follicles, leading to the formation of germinal centers.^11^ In addition to promoting the functions of B cells and CD8^+^ T cells, accumulating evidence suggests that CD4^+^ T cells also play a protective role through direct cytolytic activity during viral infections. As a distinct functional subset of T cells, CD4 CTLs are capable of directly killing infected cells through secreting GranzymeB and Perforin‐dependent lytic capacity.^12^

The aging process leads to significant changes in the immune system, resulting in reduced numbers of naïve T cells and biased T cell differentiation, making individuals more susceptible to infection and reducing vaccine effectiveness.^13^ Most recently, the aging‐associated changes in the T cell compartment have been comprehensively characterized using single‐cell RNA‐seq and single‐cell T cell receptor (TCR) sequencing. Some distinct age‐associated CD4^+^ and CD8^+^ T cell subpopulations have been identified, such as age‐associated PD1^+^TOX^+^CD8^+^ T cells. These cells make up to 60% of all CD8^+^ T cells in the spleen.^14^ However, it is unknown whether splenectomy is involved in age‐related alterations during the antiviral response.

To investigate the effects of splenectomy on the immune system, mice that had undergone splenectomy were infected with lymphocytic choriomeningitis virus (LCMV). Subsequently, the changes in T cells in peripheral lymph nodes, such as inguinal lymph nodes (ILN), axillary lymph nodes (ALN), brachial lymph nodes (BLN), cervical lymph nodes (CLN) and MLN were characterized. Our data showed that the splenectomized (splx) mice had an increase in CD8^+^GP33^+^ T cells in the MLN. Further, we provide evidence that the effector KLRG1^+^ T cells increased in the MLN of splx mice. Additionally, the number of CD4 CTLs was also increased in the peripheral lymph nodes of mice with splenectomy. Further, aged mice showed a stronger compensatory ability than adult mice, as evidenced by an increase of effector CD8^+^ T cells in all peripheral lymph nodes. Therefore, the study provides compelling evidence that T cells in MLN are crucial for individuals with splenectomy to protect against viral infections.

## 2 MATERIALS AND METHODS

### 2.1 Mice

C57BL/6J mice were purchased from Vital River Company (Beijing, China). All mice were bred and maintained in standard laboratory conditions (20 ± 2°C with a relative humidity of 50 ± 5% and a 12‐h light: 12‐h dark cycle) and were administered food and water ad libitum. All animal procedures were approved by the Animal Study Ethics Committee of Xi'an Jiaotong University and the Institutional Animal Care and Use Committee of Xi'an Jiaotong University.

### 2.2 Splenectomy operation

As H. Chen et al described.^15^ Mice were anaesthetised through anaesthesia inhalation. After shaving off the hair on left body, a 0.5 cm vertical incision was made on the skin at the interface of abdomen and back. The peritoneum was cut to expose the spleen. Splenic artery, veins and nerves were ligated, the spleen was cut off and the peritoneum and skin were sutured. Animals were kept at 37°C until they woke up. The mice were observed daily in the following 2 weeks. Sham operation (sham) group underwent the same procedure expect vessels ligation and spleen removal. All mice were maintained in the same conditions.

### 2.3 LCMV and LM‐GP33 infection

As described previously.^16^ LCMV‐Armstrong was propagated in BHK‐21 cells. The viral titers were determined using plaque assays in Vero cells. In the LCMV infection model, mice were infected with 2 × 10^5^ plaque‐forming units (PFUs) of LCMV‐Armstrong intraperitoneally. The lymphocytes and virus lode were analysed on the indicated days after infection. L. monocytogenes expressing GP33 (LM‐GP33) strain was grown to mid‐log phase at 37°C in a culture of brain heart infusion (BHI) agar and frozen at −70°C. The mice were infected with 1 × 10^6^ (lethal) colony‐forming units (CFU) of LM‐GP33 intravenously. The bacteria load was measured in the liver of survived mice day7 after infection.

### 2.4 Antibodies and reagents

The Abs used are as follows: APC/Cy7 anti‐mouse CD4 (clone GK1.5), APC anti‐mouse CD4 (clone GK1.5), FITC anti‐mouse CD8a (clone 53–6.7), APC/Cy7 anti‐mouse CD8 (clone 53–6.7), PE/Cy7 anti‐mouse/human CD44 (clone IM7), APC anti‐mouse CD62L (clone MEL‐14), APC anti‐mouse/human KLRG1(MAFA) (clone 2F1/KLRG1), PE/Cy7 anti‐mouse IFNγ (clone XMG1.2), PE anti‐mouse Perforin (clone S16009A), Fixation Buffer(Cat # 420801) and Intracellular Staining Perm Wash Buffer(Cat # 421002). All reagents were purchased from BioLegend. FITC anti‐mouse GranzymeB (clone NCZB) was purchased from eBioscience. RNAprep pure Cell/Bacteria Kit RNAprep pure (Cat # DP430) was purchased from TIANGEN BIOTECH (Beijing) CO, LTD. PB anti‐mouse GP33 tetramer was obtained from NIH Tetramer Core Facility.

### 2.5 Fluorescence‐activated cell sorting (FACS) analysis

Single‐cell suspensions were prepared from ILN, BLN, ALN, CLN, and MLN, and stained with anti‐CD4, CD8, GP33, CD44, CD62L, and KLRG1 Abs in the dark at 4°C for 30 min. After washing with cold FACS buffer (1× PBS supplemented with 2% FBS), cells were analysed using CytoFLEX flow cytometer (Beckman Coulter, Brea, CA, USA). CytExpert 2.4 was used for data analysis.

For cytokine analysis, lymphocytes from lymph nodes were stimulated in vitro with PMA/Ionomycin in the presence of Brefeldin A (BioLegend) and monensin (BioLegend) for 4 h in 37°C, 5% CO_2_ environment. Cells were washed and stained with anti‐CD4, CD8, and GP33 antibodies. After a 30‐min incubation and FACS wash, cells were fixed and permeabilized using Fixation/ Permeabilization buffer (BioLegend), and stained with IFNγ, Perforin, and GranzymeB antibodies and FACS analysis.

### 2.6 Quantitative polymerase chain reaction (qPCR)

RNAprep pure Cell/Bacteria Kit RNAprep pure was used to extract total RNA from liver whole cells following the manufacturer's instruction. cDNA was synthesized by the cDNA synthesis kit (TOYOBO). qPCR was performed on StepOnePlus™ Real‐Time PCR System (ABI) using SYBR Green RT‐qPCR Mastermix (GenStar).

### 2.7 Statistical analysis

Data are presented as mean ± standard deviation (SD). Two‐tailed Student's *t*‐test was used for all statistical calculations. Statistical significance was analysed using the GraphPad Prism 7.0 (USA, GraphPad Software Inc.) statistical program. The level of significance is indicated as follows: *p < 0.05, **p < 0.01, ***p < 0.001 and ****p < 0.0001.

## 3 RESULTS

### 3.1 Splenectomy had no effect on T cell homeostasis in the lymph nodes

**FIGURE 1:**
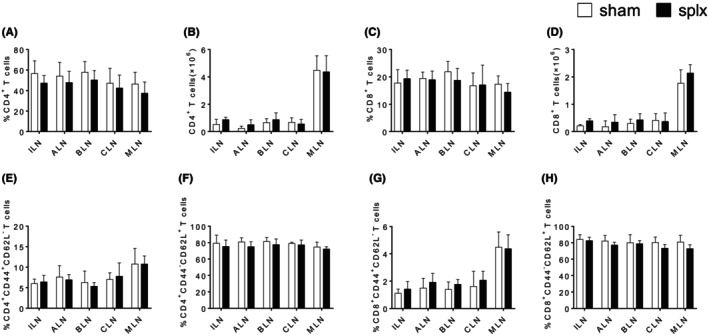
Effect of splenectomy on T cell populations in peripheral lymph nodes. (A, B) The percentage and number of CD4+ T cells in the ILN, ALN, BLN, CLN and MLN of sham and splx mice. (C, D) The percentage and number of CD8+ T cells in the ILN, ALN, BLN, CLN and MLN of sham and splx mice. (E, F) The percentage of naïve and effector CD4+ T cells in the ILN, ALN, BLN, CLN and MLN of sham and splx mice. (G, H) The percentage of naïve and effector CD8+ T cells in the ILN, ALN, BLN, CLN and MLN of sham and splx mice. Data are representative of at least three independent experiments.

To fully characterize the effects of splenectomy on T cell population, we utilized the splenectomy mouse model to analyse the proportion of T cells in peripheral lymph nodes. Two weeks after the surgery, we collected the peripheral lymph nodes, including ILN, ALN, BLN, CLN, MLN, from mice that underwent sham operation and mice that underwent splenectomy. As shown in Figure 1A–D and Figure S1A, the populations of CD4^+^ and CD8^+^ T cells were observed. The percentages and numbers of CD4^+^ and CD8^+^ T cells in the splenectomy group were similar to those in the sham operation group in all peripheral lymph nodes. Additionally, T cell sub‐populations were further identified mainly by CD62L and CD44 expression. Naïve T cells were characterized as CD62L^+^CD44^−^, while effector memory T cells (TEM) were characterized as CD62^−^CD44^+^. Similarly, there were no abnormalities in differentiation for CD4^+^ and CD8^+^ T cells in any of the peripheral lymph nodes after splenectomy (Figure 1E–H and Figure S1B‐C). The data suggest that splenectomy did not affect T cell homeostasis in peripheral lymph nodes.

### 3.2 Effect of splenectomy on the immune response against LM and LCMV in mice

**FIGURE 2:**
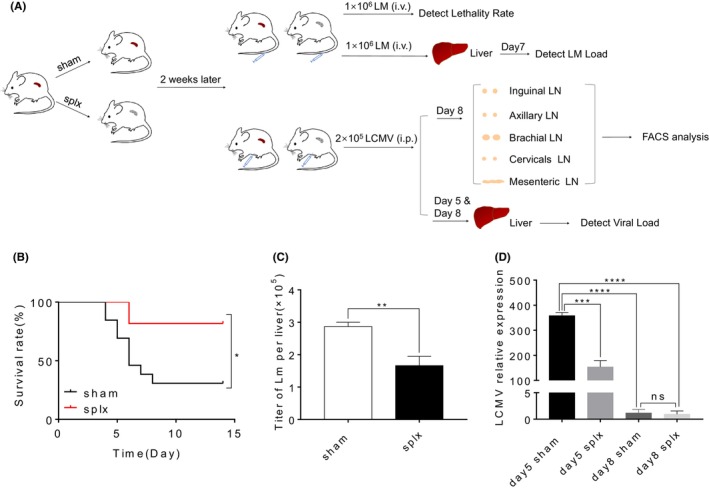
Splx mice have lower LM and LCMV load. (A) Experimental schematic of splenectomy surgery to determine the LM and LCMV clearance. Bacterial loads were determined in the liver 7 days after infection. Viral loads were determined in the liver 5 and 8 days after infection. (B) Survival curves for the mice after LM infection (1 × 10^6^ CFU per mouse; *n* = 8 mice per group). (C) The numbers of bacterial colonies per 100 mg liver from sham and splx mice. (*n* = 3 mice per group). (D) Relative expression of LCMV in the liver of mice in day 5 and day 8 after infection. (*n* = 5 mice per group). Data are representative of at least three independent experiments. *p < 0.05, **p < 0.01, ***p < 0.001 and ****p < 0.0001.

To further investigate the impact of splenectomy on T cell immunity to infection, we infected sham mice and splx mice with 1 × 10^6^ CFU of LM‐GP33 2 weeks after surgery (Figure 2A). We found that mice that underwent splenectomy showed an increased survival rate during primary infection (Figure 2B). Consistently, the severity of pathogen infection decreased in mice that underwent splenectomy, as evidenced by the lower LM‐GP33 titre in the liver tissues (Figure 2C). In addition, 2 × 10^5^ PFU of LCMV‐Armstrong were used to infect sham and splx mice (Figure 2A). Also, a decreased severity of virus infection was detected in mice that underwent splenectomy. This was confirmed by qPCR, which showed a lower viral load in the liver tissues of the splenectomy mice (Figure 2D). Together, splenectomy did not display an impairment of immune defence against LM and LCMV.

### 3.3 An increase of antigen‐specific CD8+ T cells in the MLN of splx mice infected with LCMV

**FIGURE 3:**
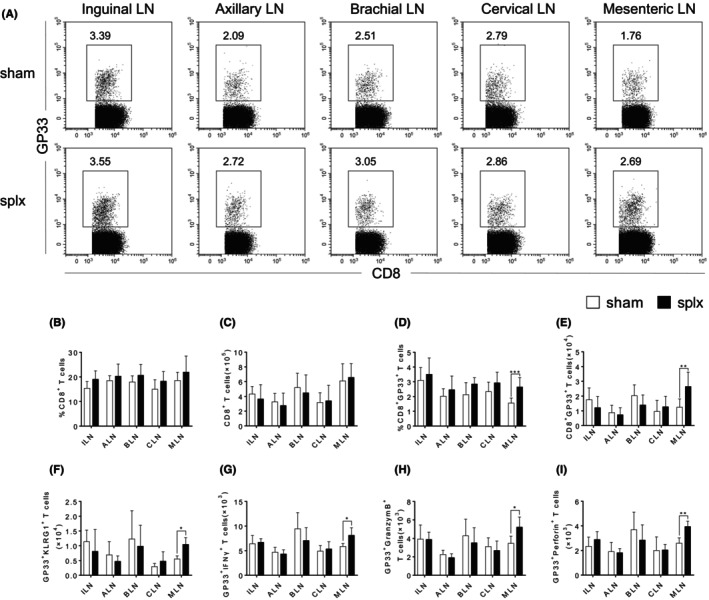
An increase of antigen‐specific CD8+ T cells in the MLN of splx mice upon infection. Sham and splx mice were challenged with 2 × 105 CFU of LCMV. GP33‐specific CD8+ T cells in both groups of mice were measured by flow cytometry. (A) Representative FACS plots of GP33‐specific CD8+ T cells. (B, C) The percentage and number of CD8+ T cells in the ILN, ALN, BLN, CLN and MLN of sham and splx mice. (D, E) The percentage and number of GP33‐specific CD8+ T cells in the ILN, ALN, BLN, CLN and MLN of sham and splx mice. (F) The number of GP33‐specific CD8+KLRG1+ T cells in the ILN, ALN, BLN, CLN, and MLN of sham and splx mice. (G) The number of GP33‐specific CD8+IFNγ+ T Cells in the ILN, ALN, BLN, CLN, and MLN of sham and splx mice. (H) The number of GP33‐specific CD8+GranzymeB+ T cells in the ILN, ALN, BLN, CLN and MLN of sham and splx mice. (I) The number of GP33‐specific CD8+Perforin+ T cells in the ILN, ALN, BLN, CLN and MLN of sham and splx mice. Data are representative of at least three independent experiments. *p < 0.05, **p < 0.01, ***p < 0.001.

We next sought to explore the compensatory function of T cells from the peripheral lymph nodes in mice that underwent splenectomy during the immune response by using the LCMV infection model. Given the significance of CD8^+^ T cells in the antiviral response, we first analysed the percentages and absolute numbers of antigen‐specific CD8^+^ T cells in response to LCMV infection. Although there were no aberrations in the percentages and numbers of total CD8^+^ T cells in all the peripheral lymph nodes (Figure 3B,C and Figure S2A), our data showed that the splx mice had a significant increase in both the percentages and absolute numbers of CD8^+^GP33^+^ T cells in the MLN on day 8 (Figure 3A,D,E). Further examination of cell surface phenotype of CD8^+^ T cells revealed that the frequency of terminally differentiated effector (TE) CD8^+^ T cells, characterized by KLRG1, was increased in the MLN of splx mice (Figure 3F). Furthermore, CD8^+^GP33^+^ T cells in the MLN exhibited an increased expression of IFNγ, GranzymeB and Perforin on day 8 (Figure 3G,H and Figure S3A‐C). Therefore, these data demonstrate that CD8^+^ T cells in the MLN significantly compensate for the immune function of the spleen to protect against infection.

### 3.4 Increased CD4 CTLs in the peripheral lymph nodes of splx mice infected with LCMV

**FIGURE 4:**
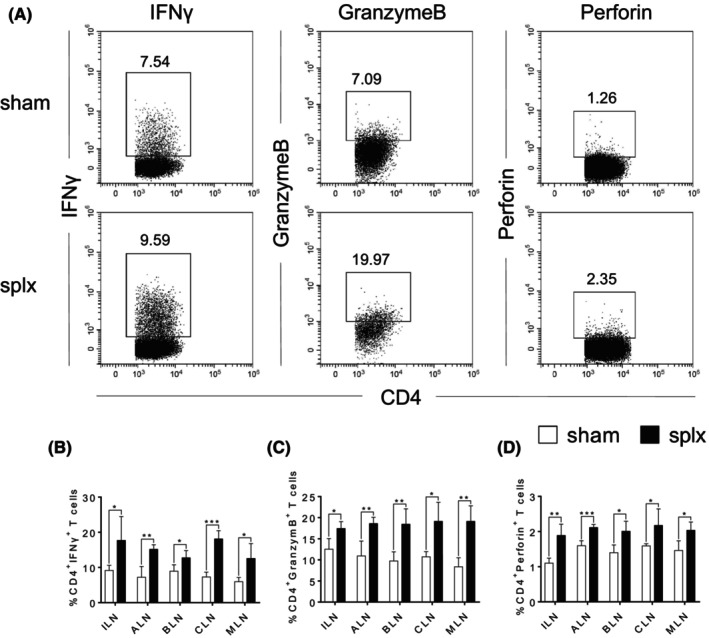
Increased proportion of CD4 CTLs in peripheral lymph nodes of splx mice upon infection. Sham and splx mice were challenged with 2 × 10^5^ CFU of LCMV. CD4^+^ T cells in both groups of mice were measured by flow cytometry. (A) Representative FACS plots of CD4^+^ T cells in MLN of sham and splx adult mice infected by LCMV. (B) The percentage of CD4^+^IFNγ^+^ T cells in the ILN, ALN, BLN, CLN and MLN of sham and splx mice. (C) The percentage of CD4^+^GranzymeB^+^ T cells in the ILN, ALN, BLN, CLN and MLN of sham and splx mice. (D) The percentage of CD4^+^Perforin^+^ T cells in the ILN, ALN, BLN, CLN and MLN of sham and splx mice. Data are representative of at least three independent experiments. *p < 0.05, **p < 0.01, ***p < 0.001.

Accumulating evidence suggests that effector CD4^+^ T cells have direct protective roles against viral pathogens through the production of IFNγ and cytolytic activity mediated by GranzymeB and Perforin.^10^ Therefore, we further analysed the percentages and absolute numbers of CD4^+^ T cells in response to LCMV infection. Still, there were no abnormalities in the percentages of CD4^+^ T cells in any of the peripheral lymph nodes (Figure S2A). However, we found that the splx mice had a significant increase in the expression of IFNγ, GranzymeB and Perforin in the CD4^+^ T cells from all peripheral lymph nodes (Figure 4A–D and Figure S4A‐C). Collectively, CD4 CTLs in the peripheral lymph nodes may also play a role in compensating for the immune function of the spleen to protect against infection.

### 3.5 The increase of effector CD8+ T cells and CD4 CTLs in peripheral lymph nodes of aged splx mice

**FIGURE 5:**
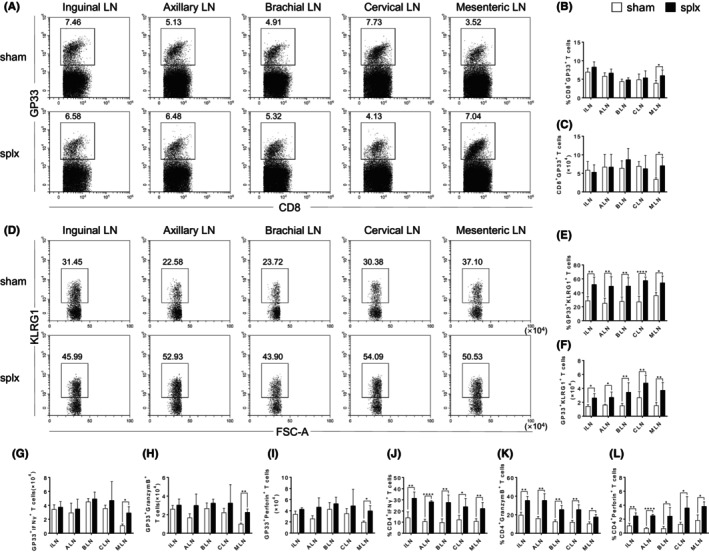
An increase of CD8^+^ and CD4^+^ T cells in peripheral lymph nodes of aged splx mice upon infection. Aged sham and splx mice were challenged with 2 × 10^5^ CFU of LCMV. CD8^+^ and CD4^+^ T cells in both mice were measured by flow cytometry. (A) Representative FACS plots of GP33‐specific CD8^+^ T cells. (B, C) The percentage and number of CD8^+^GP33^+^ T cells in the ILN, ALN, BLN, CLN and MLN of aged sham and splx mice. (D) Representative FACS plots of GP33‐specific CD8^+^KLRG1^+^ T cells. (E, F) The percentage and number of GP33‐specific CD8^+^KLRG1^+^ T cells in the ILN, ALN, BLN, CLN and MLN of sham and splx mice. (G) The number of GP33‐specific CD8^+^IFNγ^+^ T cells in the ILN, ALN, BLN, CLN and MLN of sham and splx mice. (H) The number of GP33‐specific CD8^+^GranzymeB^+^ T cells in the ILN, ALN, BLN, CLN and MLN of sham and splx mice. (I) The number of GP33‐specific CD8^+^Perforin^+^ T cells in the ILN, ALN, BLN, CLN and MLN of sham and splx mice. (J) The percentage of CD4^+^IFNγ^+^ T cells in the ILN, ALN, BLN, CLN and MLN of sham and splx mice. (K) The percentage of CD4^+^GranzymeB^+^ T cells in the ILN, ALN, BLN, CLN and MLN of sham and splx mice. (L) The percentage of CD4^+^Perforin^+^ T cells in the ILN, ALN, BLN, CLN and MLN of sham and splx mice. Data are representative of at least three independent experiments. *p < 0.05, **p < 0.01, ***p < 0.001, and ****p < 0.0001.

To investigate the impact of splenectomy on age‐related changes during the antiviral response, we created a mouse model of splenectomy in aged (65 weeks) C57BL/6J mice. Then, we infected sham and splx mice with 2 × 10^5^ PFU of LCMV 2 weeks after surgery. The phenotype of antigen‐specific CD8^+^ T cells in the peripheral lymph nodes was comparable between aged mice and adult mice. However, both percentages and absolute numbers of CD8^+^GP33^+^ T cells in the MLN showed a significant increase on day 8 after the infection (Figure 5A–C). Unexpectedly, unlike the adult mice where only the MLN showed an enlarged population of TE CD8^+^ T cells, the frequencies of KLRG1^+^CD8^+^ T cells were increased in various peripheral lymph nodes of aged mice with splenectomy (Figure 5D–F). However, CD8^+^GP33^+^ T cells from aged mice displayed similar levels of IFNγ, GranzymeB and Perforin as adult mice, expect an enhancement in the MLN (Figure 5G–I and Figure S5A‐C). Similarly, the expression of IFNγ, GranzymeB and Perforin in CD4^+^ T cells from aged mice was also increased in all peripheral lymph nodes (Figure 5J–L and Figure S6A‐C). These data demonstrate that the lymph nodes of aged mice may exhibit a stronger compensatory effect than those of adult mice.

## 4 DISCUSSION

Splenectomy may lead to weakened immune function in patients, making them more susceptible to severe infections for life. This includes an increased risk of acute fulminant infections (OPSI), sepsis and meningitis.^3^, ^17^ Lymph nodes, like the spleen are the part of the immune system and may help to restore immune function. However, there is limited research on the status and function of T cells in the lymph nodes after splenectomy. This study used a well‐established splenectomy model to examine the phenotype and function of CD8^+^ and CD4^+^ T cells in various peripheral lymph nodes during both homeostasis and viral infection scenarios.

Although the spleen and lymph nodes are both secondary lymphoid organs, they have different structure and function. For example, the spleen lacks afferent lymphatic vessels; therefore, so all antigens are delivered through the bloodstream, making it important for clearing pathogens from the blood.^18^, ^19^ However, it is not clear whether peripheral lymph nodes can compensate for the spleen's response to infection after splenectomy. Using splx mice, we found that the percentage and number of CD8^+^ and CD4^+^ T cells in various peripheral lymph nodes did not alter, and T cell subpopulations did not change either. This proves that splenectomy does not affect T cell homeostasis in the peripheral lymph nodes. Next, mice that had undergone splenectomy were infected with bacteria and viruses to assess their resistance to infection. Unexpectedly, splx mice showed a higher survival rate and lower bacterial load in their livers when infected with LM‐GP33. Similar results were also observed in the LCMV infection model. Several studies indicate that LCMV and LM may amplify in the spleen due to the intact structure of spleen, there exists a possibility that lower LCMV virus and LM bacteria load may be contributed by less production of virus or bacteria in splenectomy mice.^20^, ^21^ Of note, our data found that T cells from the lymph nodes play potential roles in compensating the influence of splenectomy on immune response.

CD8^+^ T cells are an indispensable force in efficiently controlling viral infections. Effector CD8^+^ T cells can resist viral infections by directly killing virus‐infected cells and secreting antiviral cytokines, such as IFNγ.^8^, ^22^, ^23^ Therefore, we further investigate the deviations in CD8^+^ T cell phenotype and function in the peripheral lymph nodes following virus infection. We found that CD8^+^ T cells in the MLN exhibit a stronger response to viral infection. There is a higher percentage and quantity of antigen‐specific CD8^+^ cells in the MLN, as well as a higher proportion of TE population. Additionally, they have a stronger ability to secrete IFNγ, and express GranzymeB and Perforin. Therefore, CD8^+^ T cells in the MLN may partially compensate for the spleen in its antiviral function in the mice with splenectomy. The MLN primarily receive antigens from the small intestine and the proximal colon.^24^ And pathogens in the digestive tract can translocate at a fast rate to the MLN.^25^, ^26^ Future studies are required to assess the pathways through which pathogens in the blood are transferred after splenectomy.

Studies on acute and chronic infections indicate that viral clearance is associated with the presence of highly functional and specific CD4^+^ T cell responses. In contrast, chronic infections are characterized by weak and functionally impaired CD4 T^+^ cell responses.^27^, ^28^ In response to viral infection, CD4^+^ T cells primarily differentiate into Th1 cells and secrete significant quantities of IFNγ. In addition, effector CD4^+^ T cells can also protect against viral pathogens through direct cytolytic activity mediated by GranzymeB and Perforin, known as CD4 CTLs.^29^, ^30^, ^31^ Surprisingly, we found that CD4^+^ T cells in various peripheral lymph nodes could secrete more IFNγ after splenectomy. Importantly, more CD4^+^ T cells expressed high levels of GranzymeB and Perforin. Therefore, our findings highlight the significant compensatory role of CD4^+^ T cells, particularly CD4 CTLs in the immune response to splenectomy.

We also investigated the impact of splenectomy on the anti‐infection response in aged mice. Surprisingly, antigen‐specific effector CD8^+^ T cells increased in various peripheral lymph nodes of aged mice. However, the expression of cytokine genes in CD8^+^ T cells was similar between aged and adult mice, except for cytokine elevation in the MLN of aged mice. The CD4^+^ T cells in aged mice showed a similar phenotype compared to those in adult mice. Therefore, the changes in immune response in the aged individuals are much more complex and further research is required to reveal the impact of splenectomy on the immune response in future.

In summary, the study utilized a splenectomy model to explore the compensatory function of T cells in various lymph nodes in response to viral infection. We revealed the response status of CD8^+^ and CD4^+^ T cells in various lymph nodes during both homeostasis and viral infection after splenectomy. Our findings suggest that CD8^+^ T cells from the MLN and CD4^+^ T cells from various peripheral lymph nodes play a compensatory role in the immune response against viral infections in the mice with splenectomy. These findings provide new insights into understanding the changes in the immune system of patients after splenectomy.

## AUTHOR CONTRIBUTIONS

**Xiaobin Liu:** Writing – original draft (equal). **Yae Sun:** Writing – original draft (equal). **Yanhong Su:** Writing – original draft (equal). **Yang Gao:** Data curation (equal). **Tianzhe Zhang:** Data curation (equal). **Qianhao Wang:** Data curation (equal). **Xiaoran Zhang:** Writing – review and editing (equal). **Dan Zhang:** Formal analysis (equal). **Chenming Sun:** Formal analysis (equal); writing – review and editing (equal). **Jun Li:** Conceptualization (equal); writing – review and editing (equal). **Zongfang Li:** Conceptualization (equal); writing – original draft (equal). **Baojun Zhang:** Conceptualization (equal); writing – original draft (equal); writing – review and editing (equal).

## FUNDING INFORMATION

This work was supported by grants from the National Natural Science Foundation of China (82071828, to C.S. and 32170892, to B.Z.); Innovation Capability Support Program of Shaanxi Province (2024CX‐GXPT‐45, to C.S.); the Natural Science Foundation of Shaanxi Province (2021JM‐555, to J.L.); Key Research and Development Program of Shaanxi Province (2022GXLH‐01‐16, to B.Z.); and Fundamental Research Funds for the Central Universities (xtr072022002, to B.Z.).

## CONFLICT OF INTEREST STATEMENT

We declare no competing interests.

## Supporting information


Figure S1.
Figure S2.
Figure S3.
Figure S4.
Figure S5.
Figure S6.

## Data Availability

All data, models, and code generated or used during the study are available in the submitted article.

## References

[1] Lewis SM , Williams A , Eisenbarth SC . Structure and function of the immune system in the spleen. Sci Immunol. 2019;4:eaau6085. 30824527 10.1126/sciimmunol.aau6085 PMC6495537

[2] Mebius RE , Kraal G . Structure and function of the spleen. Nat Rev Immunol. 2005;5:606‐616. 16056254 10.1038/nri1669

[3] Tahir F , Ahmed J , Malik F . Post‐splenectomy sepsis: a review of the literature. Cureus J Med Science. 2020;12:e6898. 10.7759/cureus.6898 PMC7059871 32195065

[4] Bojesen AB , Lund A , Mortensen FV , Kirkegård J . Splenectomy and risk of COVID‐19 infection, hospitalisation, and death. Infect Dis‐nor. 2021;53:678‐683. 10.1080/23744235.2021.1921257 33939582

[5] Karrer U , Althage A , Odermatt B , et al. On the key role of secondary lymphoid organs in antiviral immune responses studied in alymphoplastic (aly/aly) and spleenless (Hox11(−/−)) mutant mice. J Exp Med. 1997;185:2157‐2170. 9182687 10.1084/jem.185.12.2157 PMC2196355

[6] Karasartova D , Gazi U , Tosun O , et al. Anti‐pneumococcal vaccine‐induced cellular immune responses in post‐traumatic Splenectomized individuals. J Clin Immunol. 2017;37:388‐396. 28488145 10.1007/s10875-017-0397-3

[7] Krishnamurty AT , Turley SJ . Lymph node stromal cells: cartographers of the immune system. Nat Immunol. 2020;21:369‐380. 32205888 10.1038/s41590-020-0635-3

[8] Channappanavar R , Zhao JC , Perlman SS . T cell‐mediated immune response to respiratory coronaviruses. Immunol Res. 2014;59:118‐128. 24845462 10.1007/s12026-014-8534-z PMC4125530

[9] Qiu ZJ , Khairallah C , Sheridan BS . A model pathogen continues to refine our knowledge of the CD8 T cell response. Pathogens. 2018;7:118‐128. 10.3390/pathogens7020055 PMC6027175 29914156

[10] Swain SL , McKinstry KK , Strutt TM . Expanding roles for CD4 T cells in immunity to viruses. Nat Rev Immunol. 2012;12:136‐148. 22266691 10.1038/nri3152 PMC3764486

[11] Kervevan JM , Chakrabarti LA . Role of CD4+T cells in the control of viral infections: recent advances and open questions. Int J Mol Sci. 2021;22:136‐148. 10.3390/ijms22020523 PMC7825705 33430234

[12] Takeuchi A , Badr MESG , Miyauchi K , Kubo M , Saito T . CRTAM instructs the CD4<SUP>+</SUP> cytotoxic T lymphocyte lineage. Eur J Immunol. 2016;46:988.

[13] Montecino‐Rodriguez E , Berent‐Maoz B , Dorshkind K . Causes, consequences, and reversal of immune system aging. J Clin Invest. 2013;123:958‐965. 23454758 10.1172/JCI64096 PMC3582124

[14] Mogilenko DA , Shpynov O , Andhey PS , et al. Comprehensive profiling of an aging immune system reveals clonal GZMK CD8 T cells as conserved Hallmark of Inflammaging. Immunity. 2021;54:99. 33271118 10.1016/j.immuni.2020.11.005

[15] Chen H , Huang N , Tian H , et al. Splenectomy provides protective effects against CLP‐induced sepsis by reducing TRegs and PD‐1/PD‐L1 expression. Int J Biochem Cell Biol. 2021;136:105970. 33774183 10.1016/j.biocel.2021.105970

[16] Jiao A , Sun C , Wang X , et al. DExD/H‐box helicase 9 intrinsically controls CD8(+) T cell‐mediated antiviral response through noncanonical mechanisms. Sci Adv. 2022;8:eabk2691. 35138904 10.1126/sciadv.abk2691 PMC8827654

[17] Leone G , Pizzigallo E . Bacterial infections following splenectomy for malignant and nonmalignant hematologic diseases. Mediterr J Hematol I. 2015;7:eabk2691. 10.4084/MJHID.2015.057 PMC4621170 26543526

[18] Cataldi M , Vigliotti C , Mosca T , Cammarota M , Capone D . Emerging role of the spleen in the pharmacokinetics of monoclonal antibodies, nanoparticles and exosomes. Int J Mol Sci. 2017;18:e2015057. 10.3390/ijms18061249 PMC5486072 28604595

[19] Kmieciak AE et al. Predicted limited redistribution of T cells to secondary lymphoid tissue correlates with increased risk of haematological malignancies in asplenic patients. Sci Rep‐Uk. 2021;11:1249. 10.1038/s41598-021-95225-x PMC8360980 34385480

[20] Kuranaga N , Kinoshita M , Kawabata T , Shinomiya N , Seki S . A defective Th1 response of the spleen in the initial phase may explain why splenectomy helps prevent a listeria infection. Clin Exp Immunol. 2005;140:11‐21. 15762870 10.1111/j.1365-2249.2005.02735.x PMC1809347

[21] Muller S et al. Role of an intact splenic microarchitecture in early lymphocytic choriomeningitis virus production. J Virol. 2002;76:2375‐2383. 11836415 10.1128/jvi.76.5.2375-2383.2002 PMC153806

[22] Fang M , Sigal LJ . Antibodies and CD8 T cells are complementary and essential for natural resistance to a highly lethal cytopathic virus. J Immunol. 2005;175:6829‐6836. 16272340 10.4049/jimmunol.175.10.6829

[23] Martin MD , Sompallae R , Winborn CS , Harty JT , Badovinac VP . Diverse CD8 T cell responses to viral infection revealed by the collaborative cross. Cell Rep. 2020;31:107508. 32294433 10.1016/j.celrep.2020.03.072 PMC7212788

[24] Houston SA , Cerovic V , Thomson C , Brewer J , Mowat AM , Milling S . The lymph nodes draining the small intestine and colon are anatomically separate and immunologically distinct. Mucosal Immunol. 2016;9:468‐478. 26329428 10.1038/mi.2015.77

[25] Corradi F , Brusasco C , Fernández J , et al. Effects of pentoxifylline on intestinal bacterial overgrowth, bacterial translocation and spontaneous bacterial peritonitis in cirrhotic rats with ascites. Dig Liver Dis. 2012;44:239‐244. 22119621 10.1016/j.dld.2011.10.014

[26] Guarner C , Runyon BA , Young S , Heck M , Sheikh MY . Intestinal bacterial overgrowth and bacterial translocation in cirrhotic rats with ascites. J Hepatol. 1997;26:1372‐1378. 9210626 10.1016/s0168-8278(97)80474-6

[27] Keoshkerian E , Hunter M , Cameron B , et al. Hepatitis C‐specific effector and regulatory CD4 T‐cell responses are associated with the outcomes of primary infection. J Viral Hepat. 2016;23:985‐993. 27558465 10.1111/jvh.12576

[28] Raziorrouh B , Sacher K , Tawar RG , et al. Virus‐specific CD4+T cells have functional and phenotypic characteristics of follicular T‐helper cells in patients with acute and chronic HCV infections. Gastroenterology. 2016;150:696. 26584604 10.1053/j.gastro.2015.11.005

[29] Barbosa CD et al. Cytotoxic CD4(+) T cells driven by T‐cell intrinsic IL‐18R/MyD88 signaling predominantly infiltrate Trypanosoma cruzi‐infected hearts. elife. 2022;11:696‐706. 10.7554/eLife.74636 PMC9236613 35670567

[30] Binder B , Thimme R . CD4 T cell responses in human viral infection: lessons from hepatitis C. J Clin Invest. 2020;130:595‐597. 31904589 10.1172/JCI133222 PMC6994135

[31] Brown DM , Dilzer AM , Meents DL , Swain SL . CD4 T cell‐mediated protection from lethal influenza: perforin and antibody‐mediated mechanisms a one‐two punch. J Immunol. 2006;177:2888‐2898. 16920924 10.4049/jimmunol.177.5.2888

