# Supplementary figures and images for: The compensatory role of T cells from lymph nodes in mice with splenectomy
Source: J Cell Mol Med. 2024 May 21;28(10):e18363. doi: 10.1111/jcmm.18363 (PMC11107144; doi:10.1111/jcmm.18363)

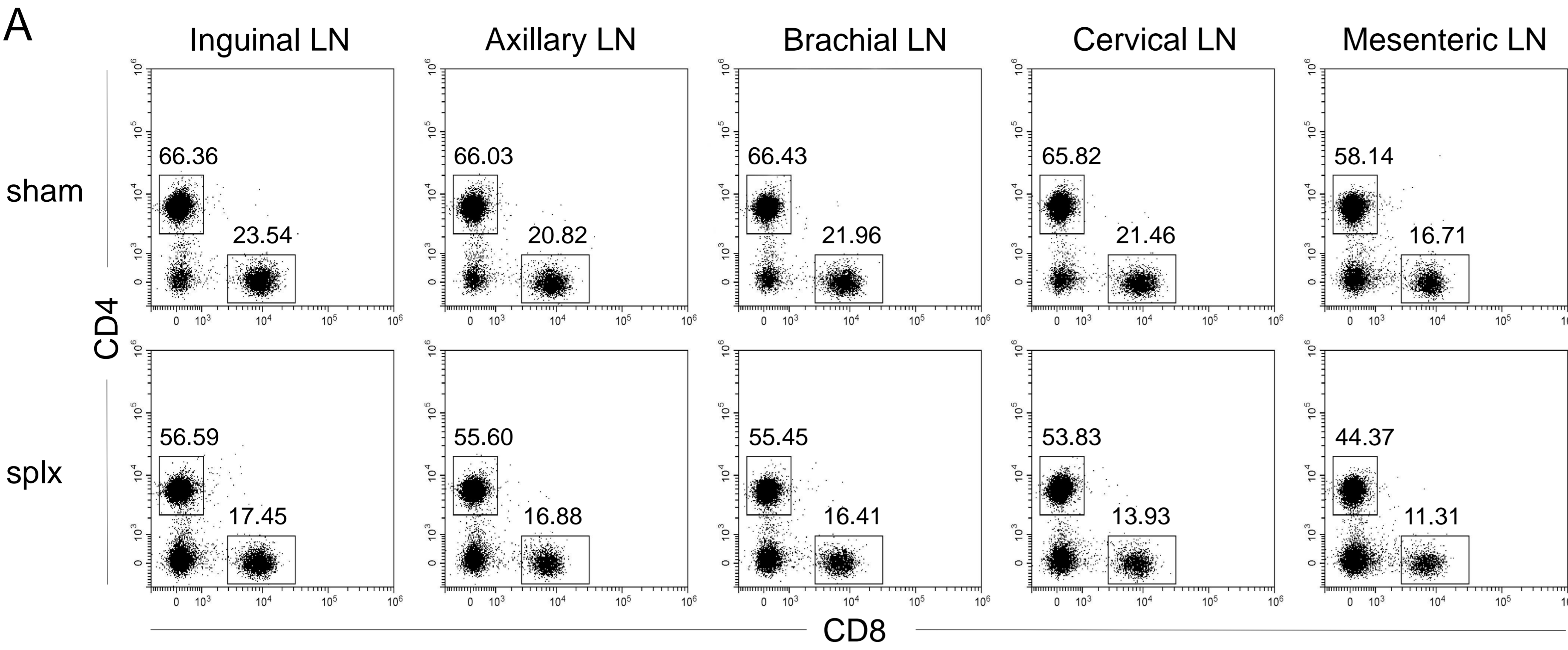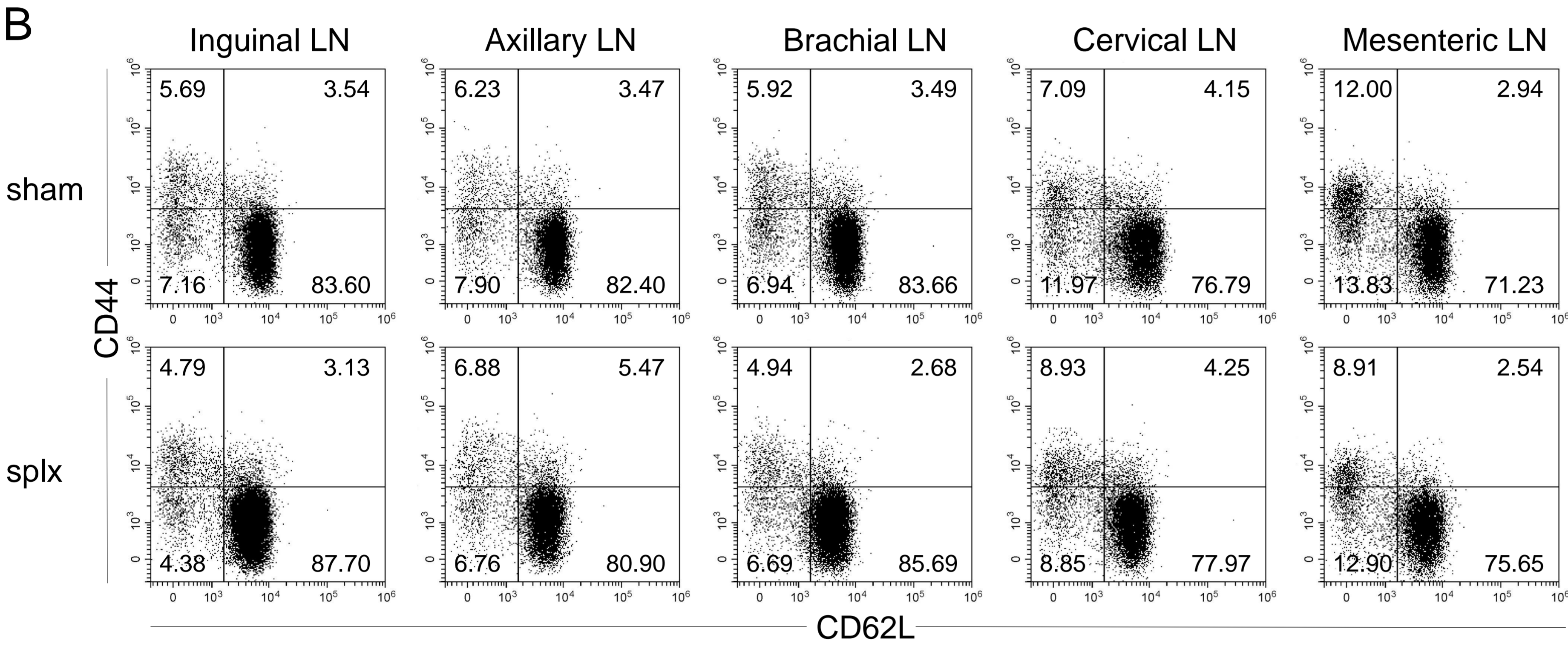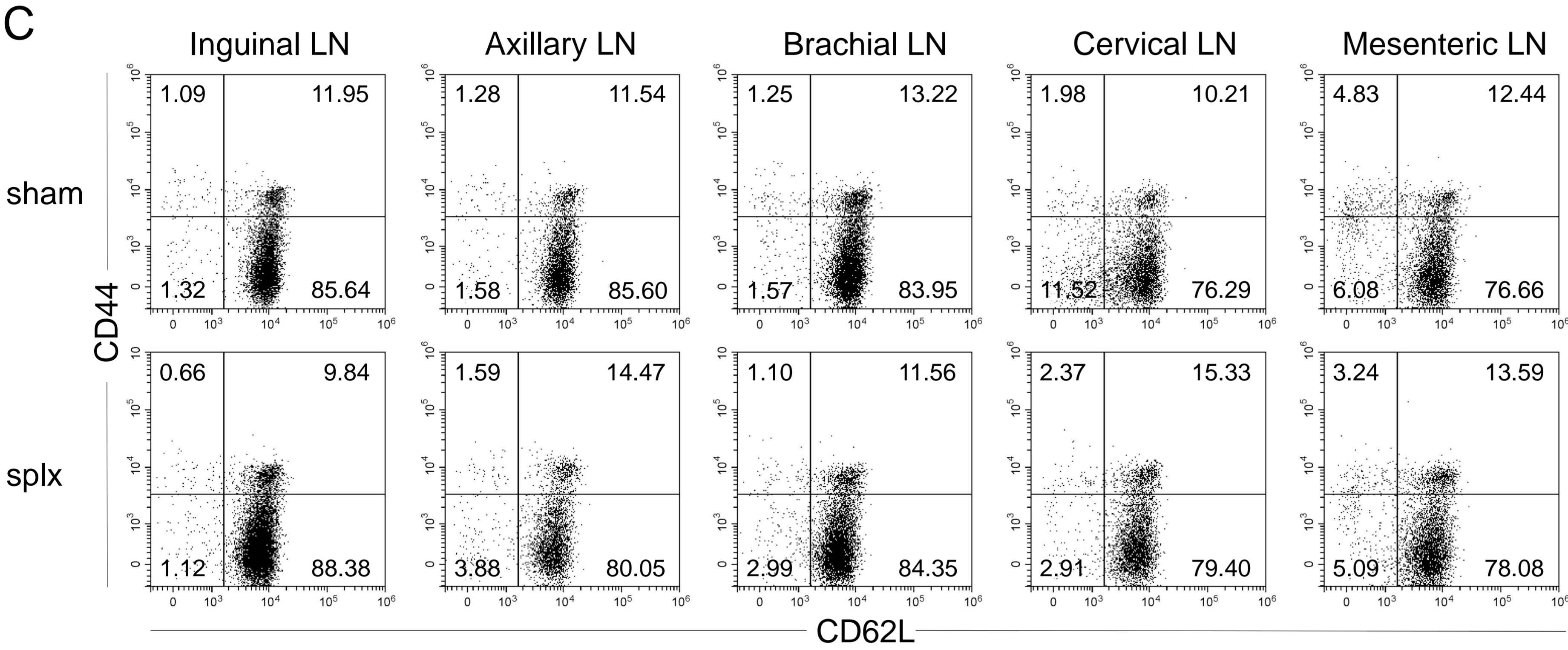

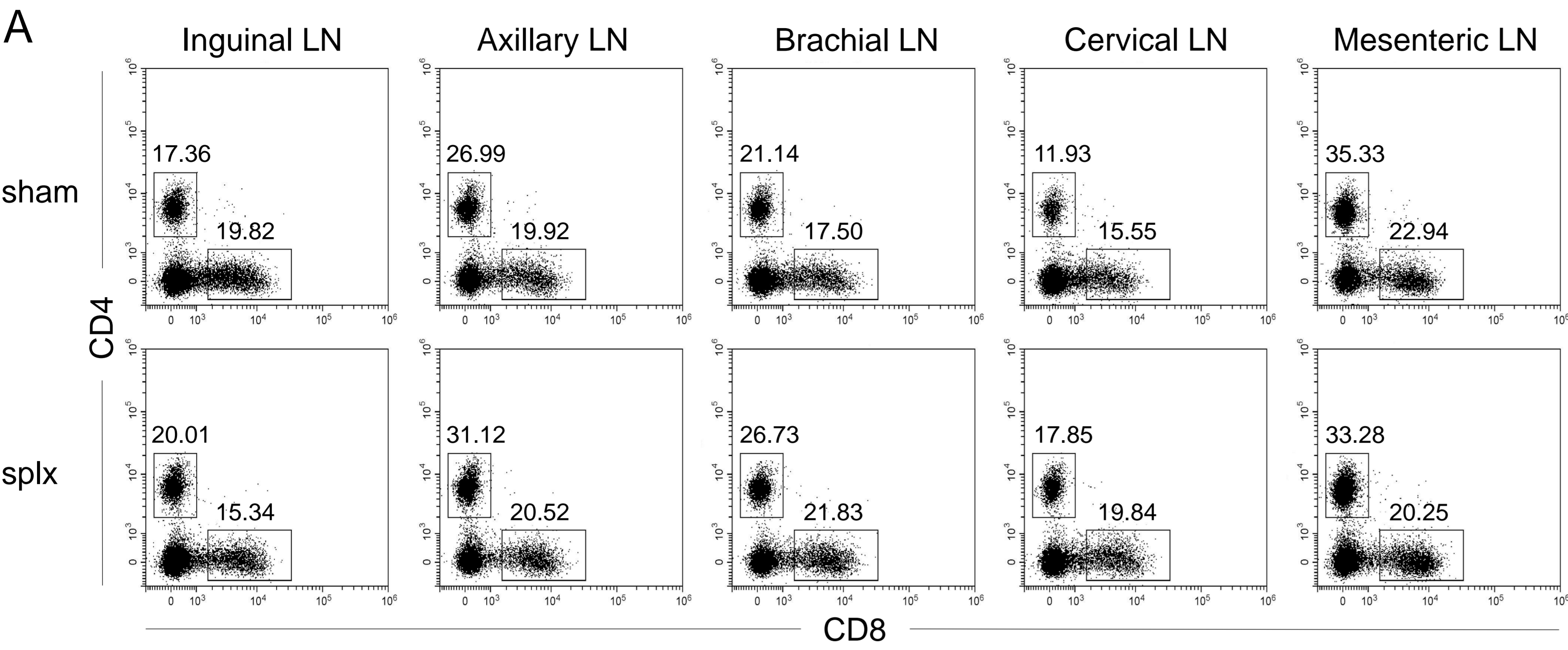

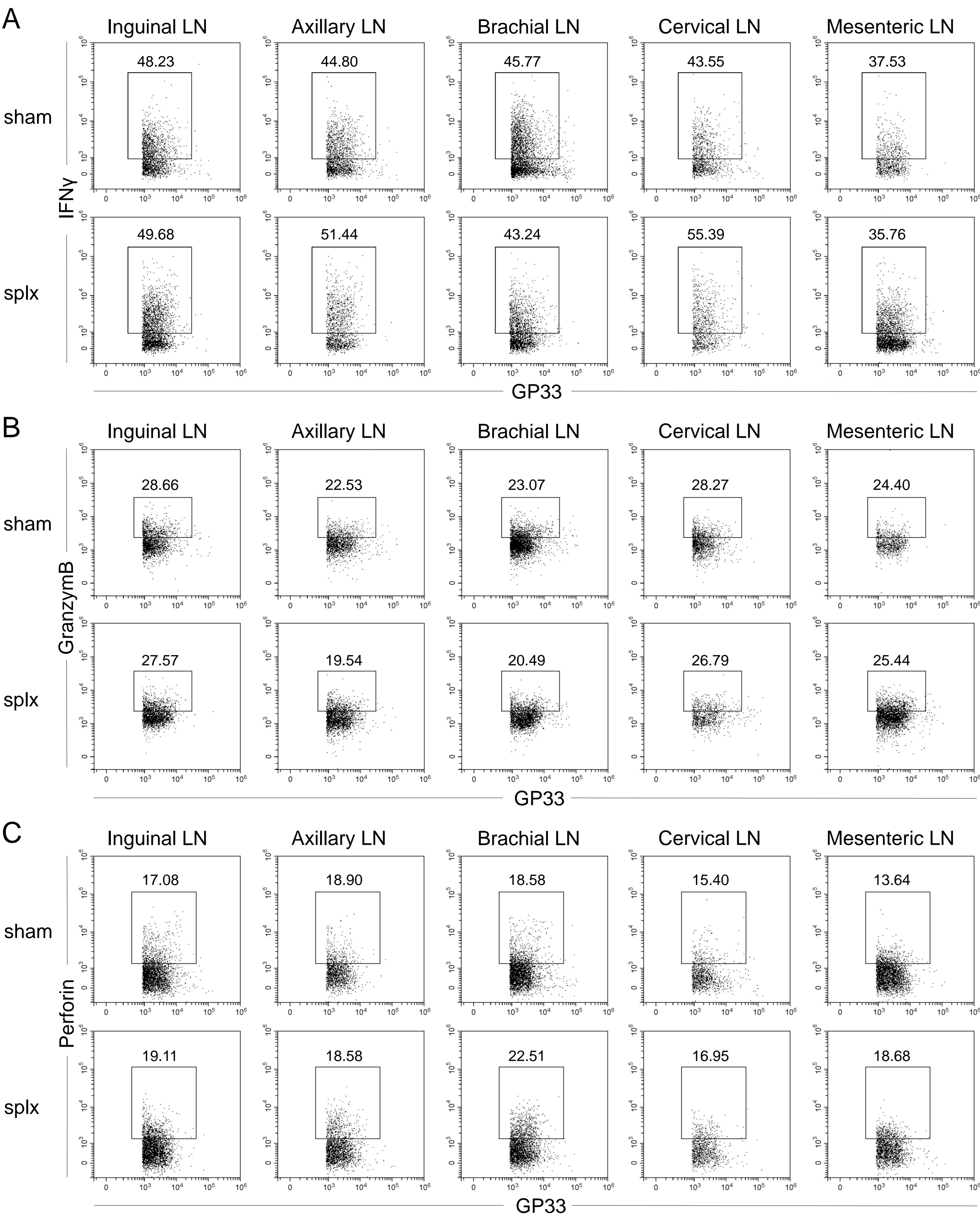

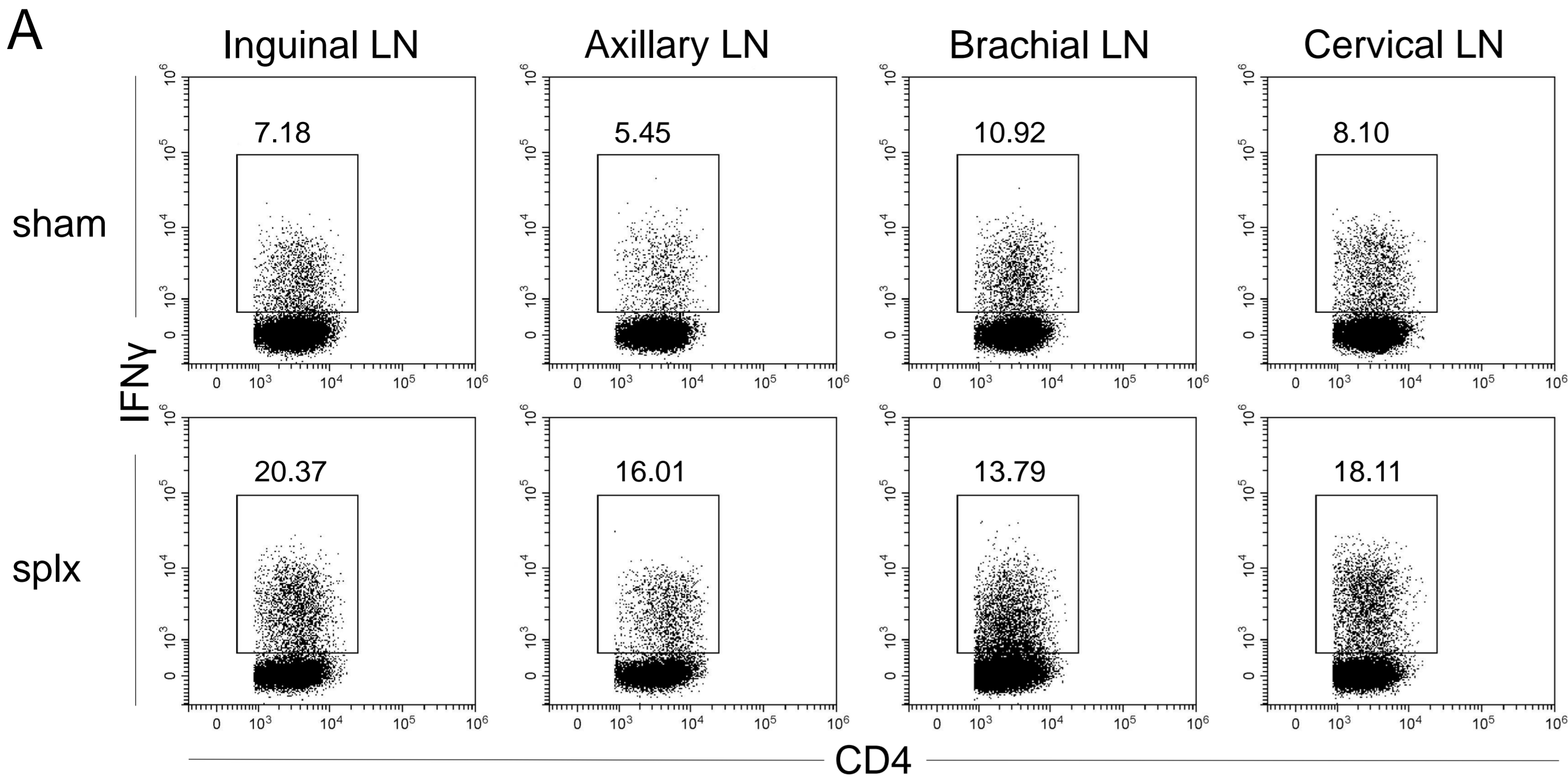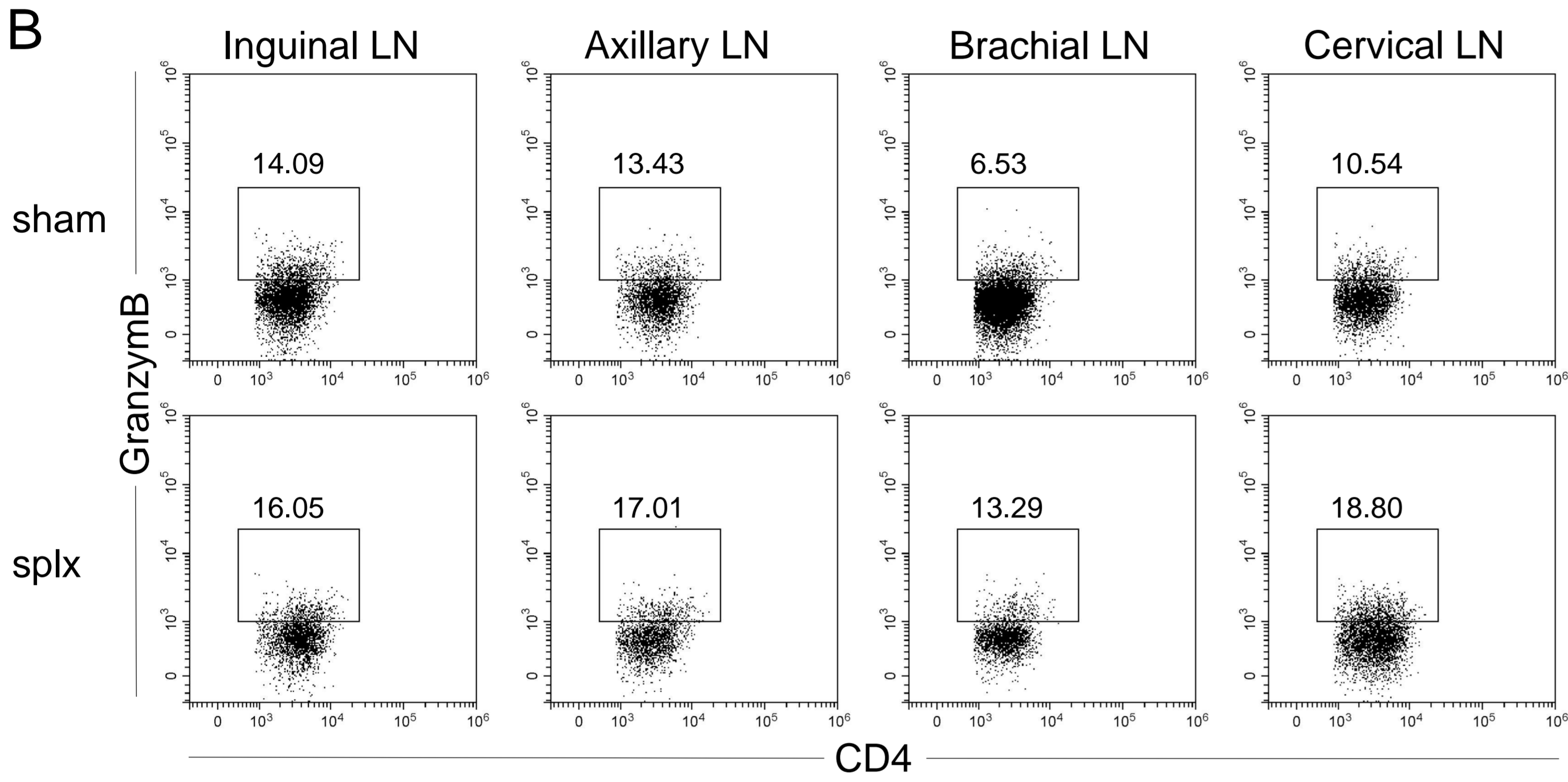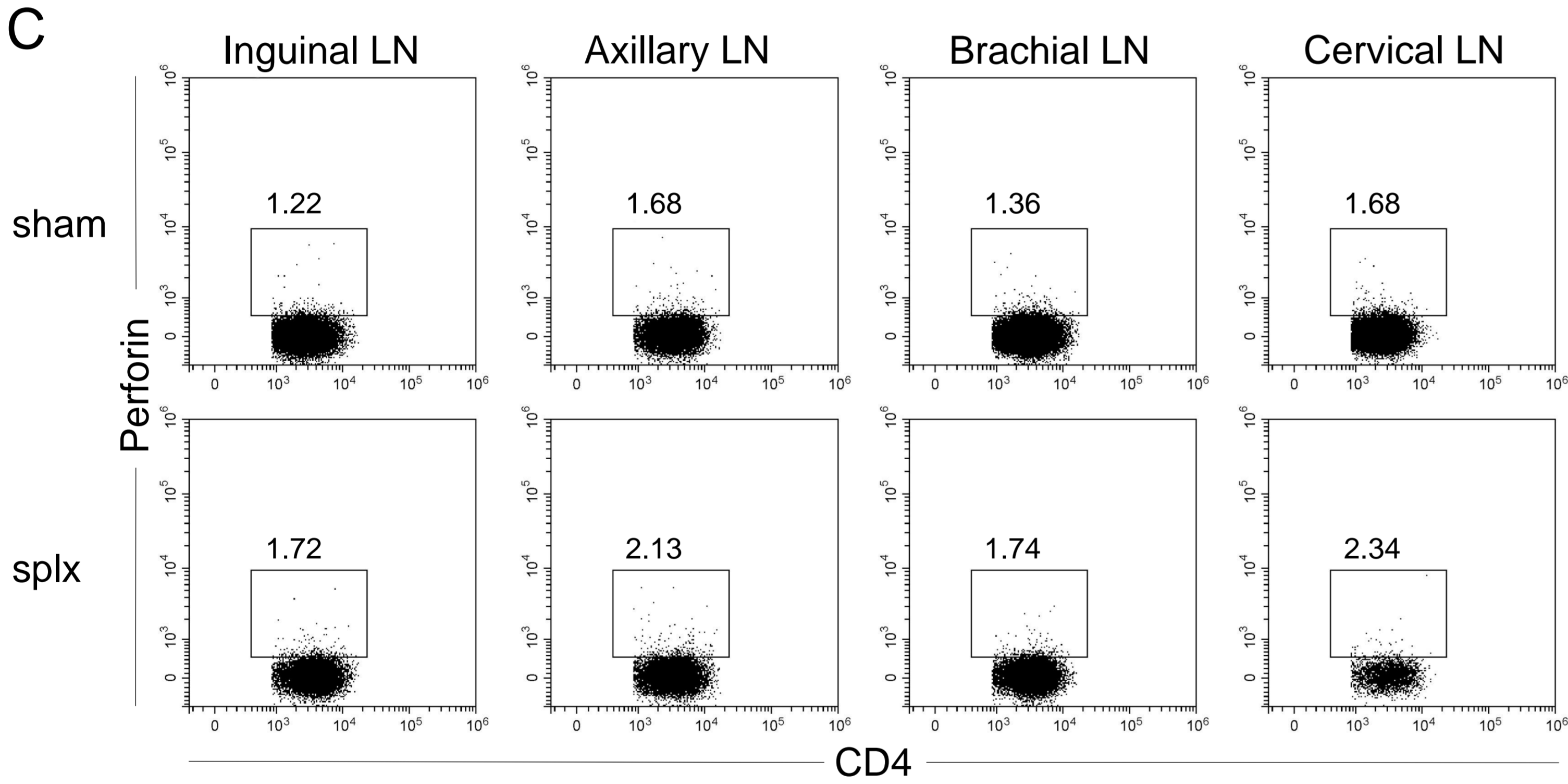

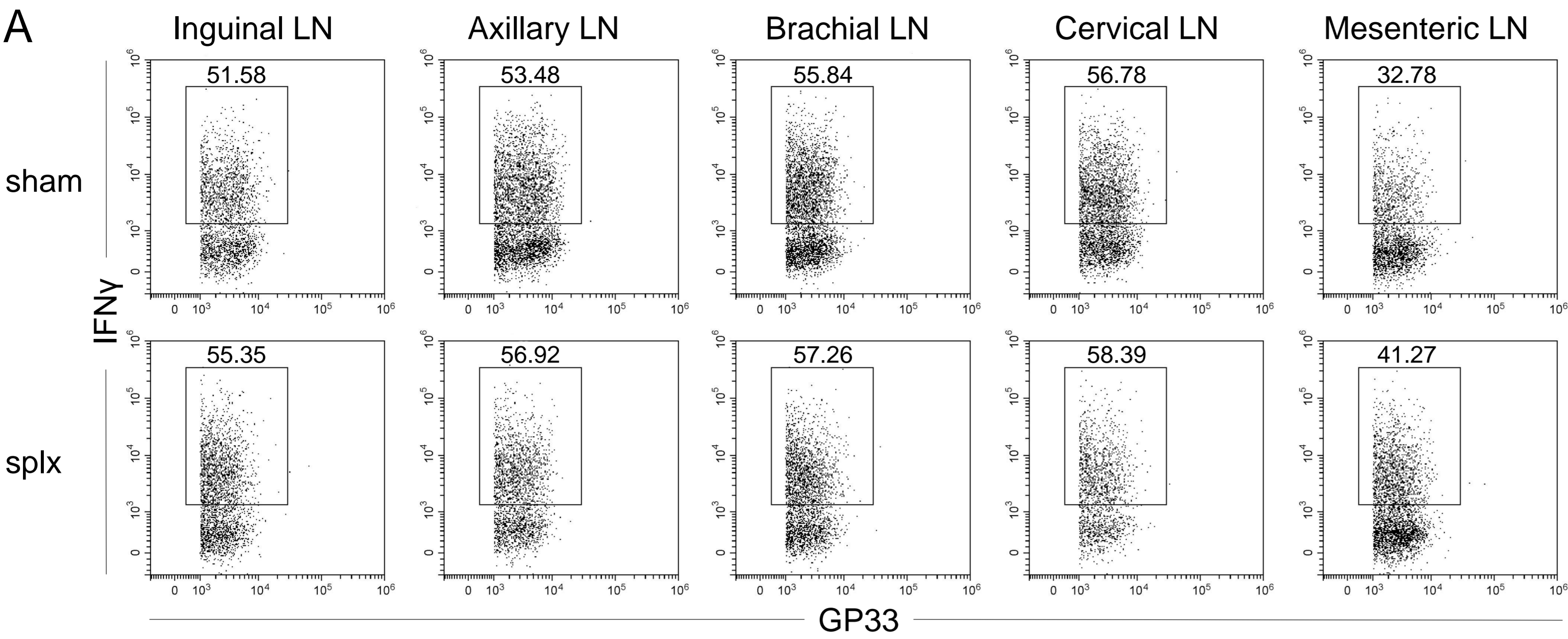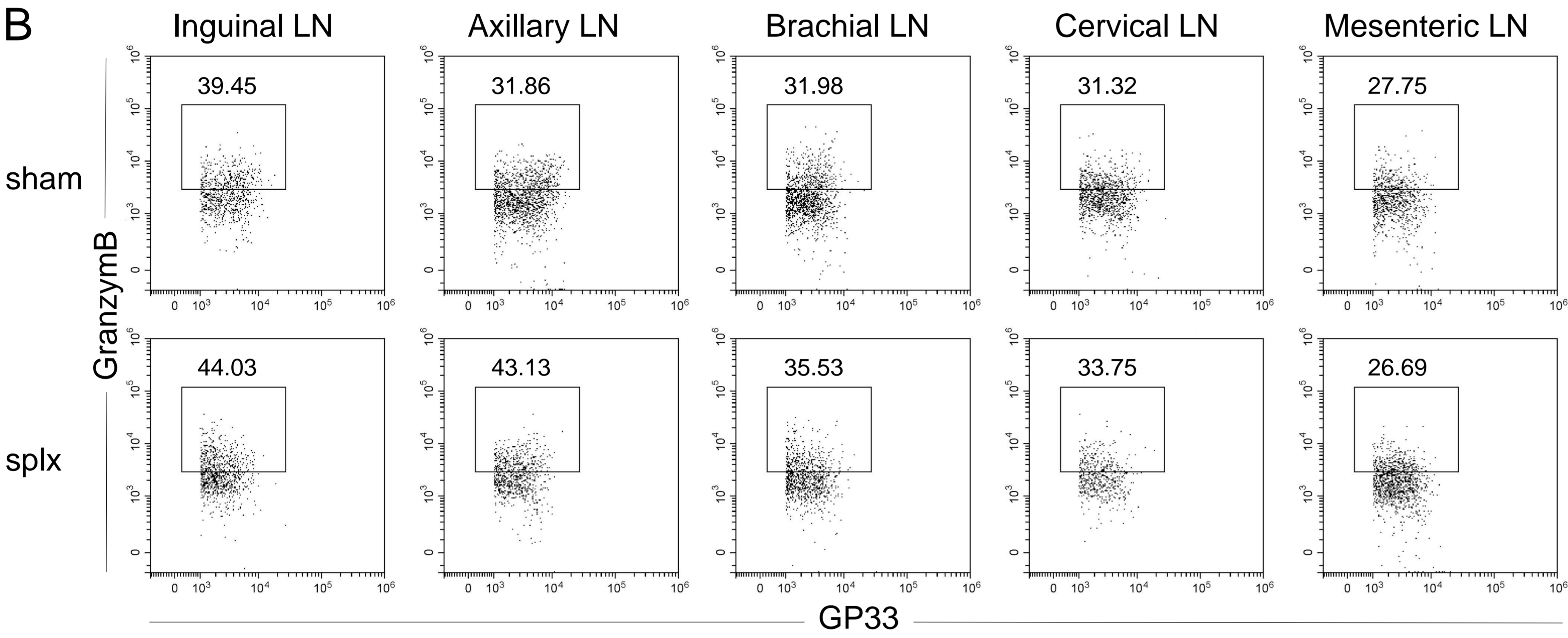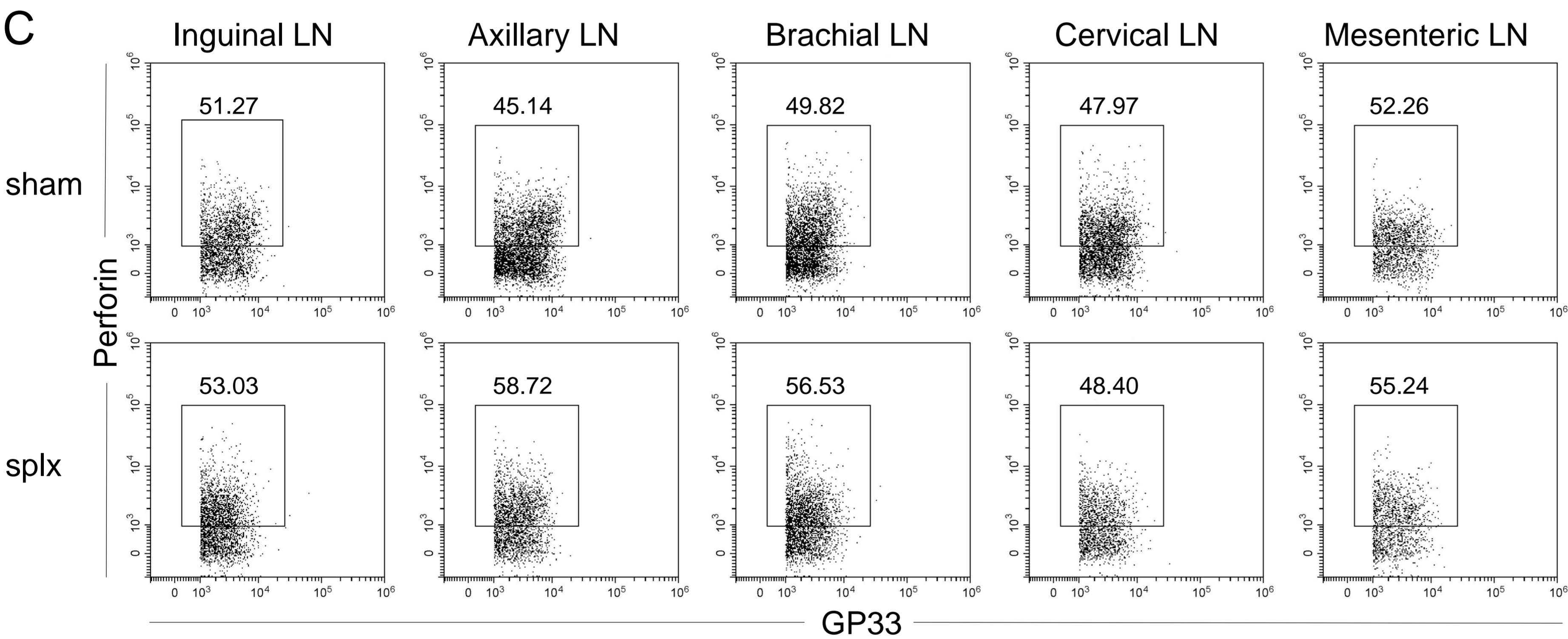

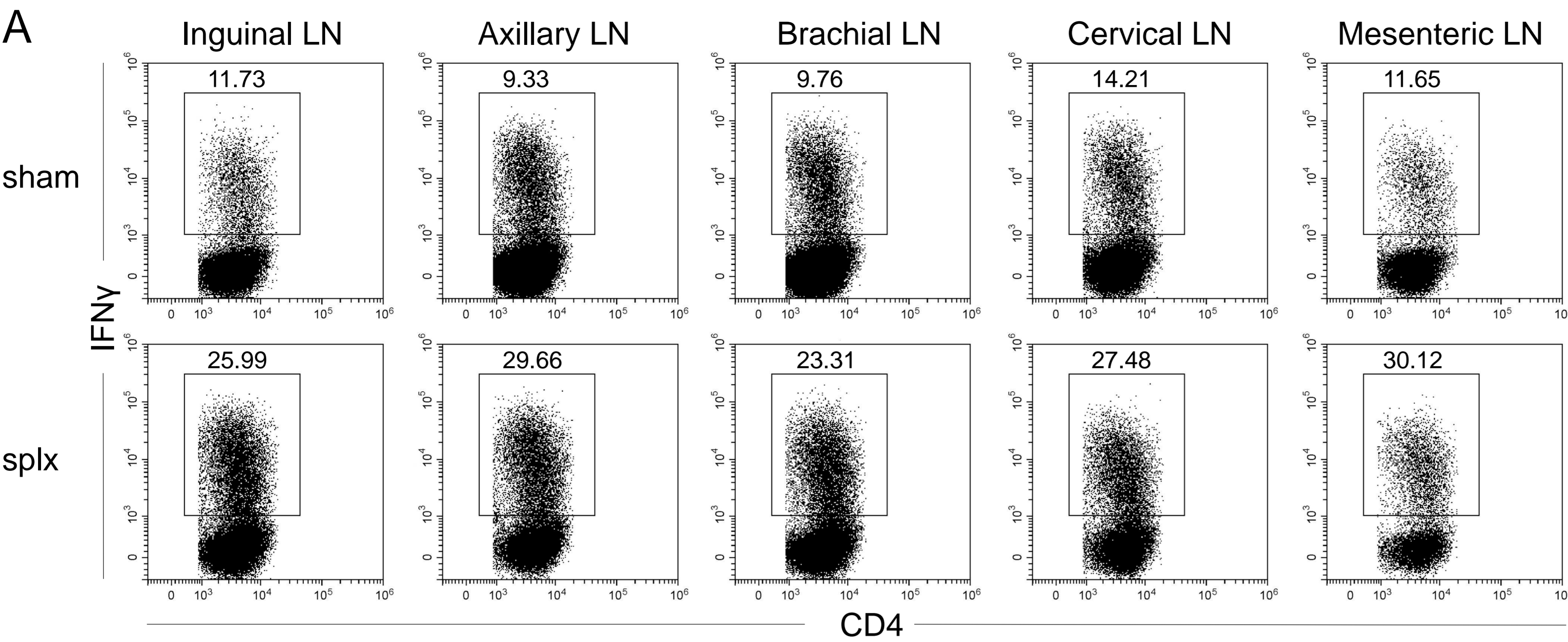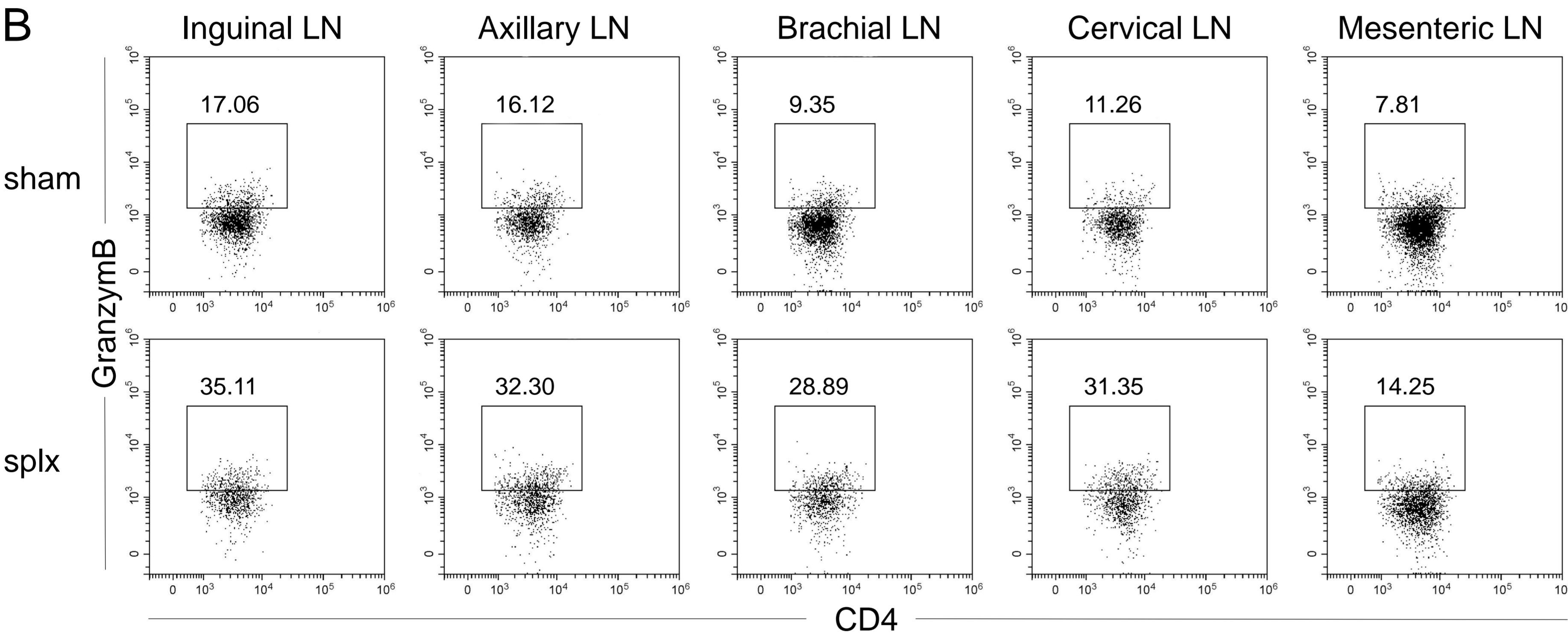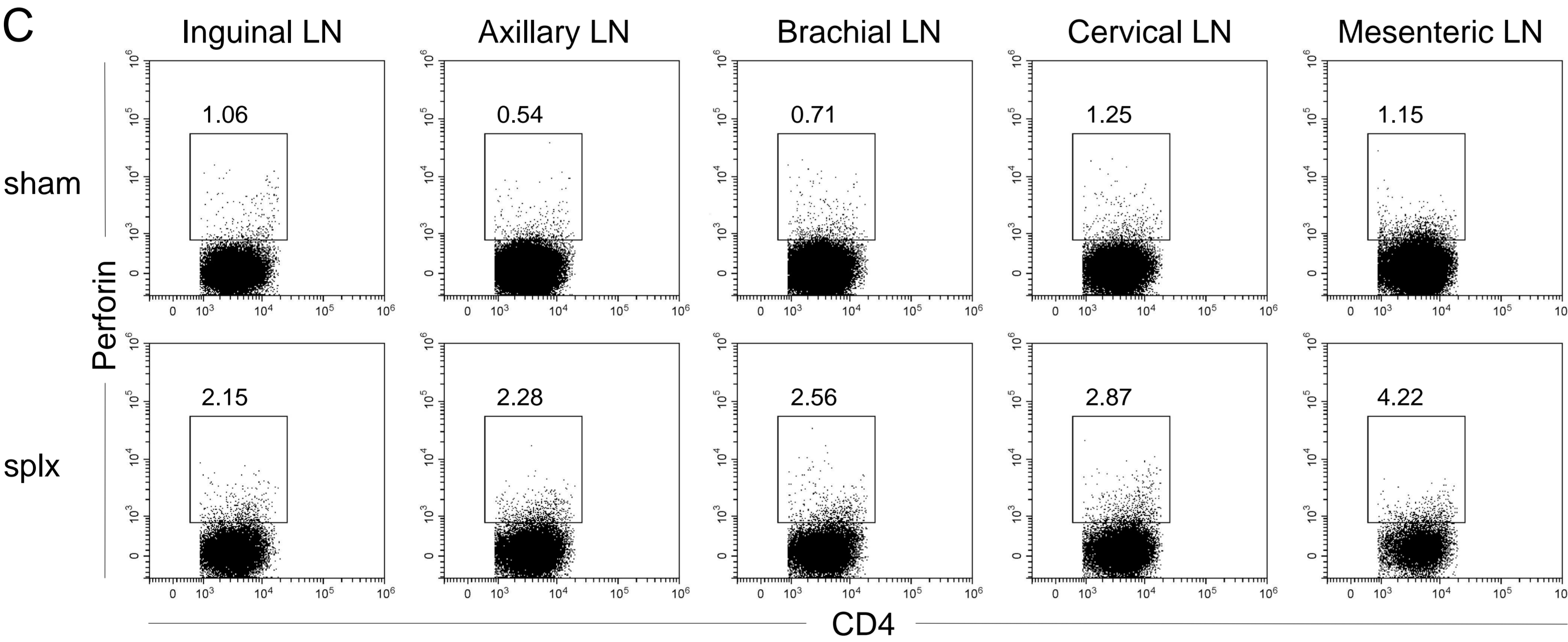

Supplement: Supplementary file 1 — Figure S1. Figure S2. Figure S3. Figure S4. Figure S5. Figure S6. [file JCMM-28-e18363-s001.pdf]
